# Successful Treatment of a Massive Desmoplastic Fibroma of the Ilium without Surgery: A Case Report with Long-Term Follow-Up

**DOI:** 10.1155/2020/5380598

**Published:** 2020-03-20

**Authors:** Hideyuki Kinoshita, Takeshi Ishii, Hiroto Kamoda, Yoko Hagiwara, Toshinori Tsukanishi, Sumihisa Orita, Kazuhide Inage, Naoya Hirosawa, Seiji Ohtori, Tsukasa Yonemoto

**Affiliations:** ^1^Department of Orthopedic Surgery, Chiba Cancer Center, 666-2 Nitonacho, Chuo-ku, Chiba 260-8717, Japan; ^2^Department of Orthopaedic Surgery, Graduate School of Medicine, Chiba University, 1-8-1 Inohana, Chuo-ku, Chiba 260-8670, Japan

## Abstract

Desmoplastic fibroma of the bone (DFB) is a notably rare, lytic, locally aggressive but nonmetastatic, primary benign bone tumor in patients less than 30 years old. As the recommended primary treatment for DFB, wide resection is preferred to curettage from the perspective of recurrence but wide resection of DFB in the pelvis such as in the acetabulum could result in greater functional loss, suggesting the need for conservative treatments. However, there is no report on long-term follow-up following conservative treatment for DFB. The present case involved a 21-year-old woman with right hip pain. Radiological evaluation revealed a massive lesion throughout the right ilium and acetabulum with partial osteolysis, cortical destruction, marginal sclerosis, slight pseudotrabeculation, and bone expansion. Open biopsy from the ilium showed the proliferation of spindle cells in an abundant collagenous matrix without atypia and mitosis, suggesting a diagnosis of DFB. Conservative treatment was selected considering the risk of greater functional loss following wide ilium resection. An evaluation 10 years after follow-up showed a partially sclerotic lesion of the ilium and the absence of pain. The current case demonstrates that conservative therapy may be effective even in some cases of aggressive DFB.

## 1. Introduction

Desmoplastic fibroma of the bone (DFB) is a notably rare, lytic, locally aggressive but nonmetastatic primary bone tumor. This tumor currently accounts for 0.06% of all bone tumors and 0.3% of benign bone tumors [1]. It occurs during the first three decades of life in over 75% of cases, with an equal male and female preponderance. The common sites of occurrence are the long tubular bones (56%), mandible (26%), and pelvis (14%) [2]. Intralesional surgical procedures for DFB such as curettage have been associated with a high rate of recurrence, which has been estimated to range from 50% to 72% [3]. Although wide resection is preferable for better results with only a 17% recurrence [4], it is associated with poor functional prognosis in massive pelvis tumors including sites such as the acetabulum. Although conservative treatment without surgery is also a treatment option when considering age, sex, and greater functional loss after wide resection, case reports on long-term follow-ups of DFB without surgery are uncommon. Here, we present a case of DFB involving the ilium, with good outcomes 10 years after treatment without surgery.

## 2. Case Presentation

**Figure 1:**
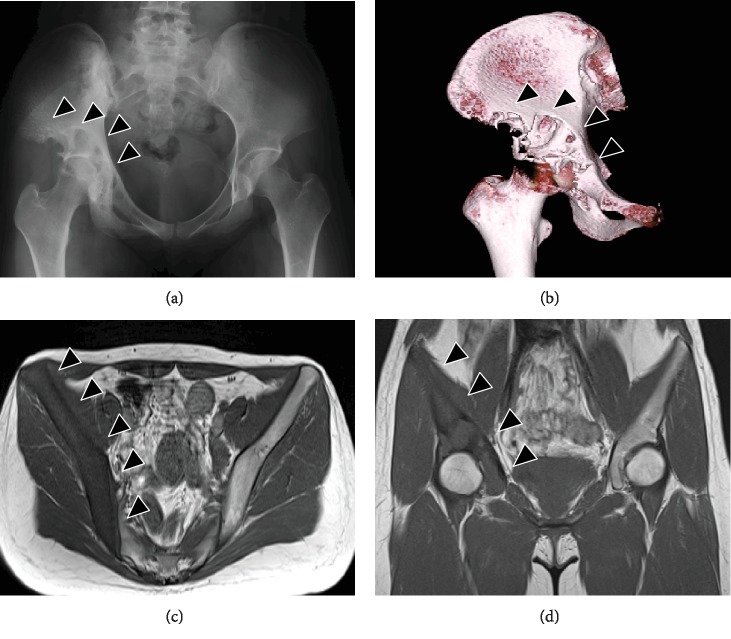
(a, b) Radiography and three-dimensional computed tomography (3D-CT) reconstruction showed an osteolytic lesion of the right ilium and acetabulum with cortical destruction, marginal sclerosis, slight pseudotrabeculation, and bone expansion (arrowhead). (a) Radiograph. (b) 3D-CT. (c, d) Magnetic resonance imaging revealed a massive tumor lesion throughout the right ilium; T2-weighted images of the ilium lesion showed low-signal intensity compared to muscle (arrowhead). (c) Axial view. (d) Coronal view.

**Figure 2:**
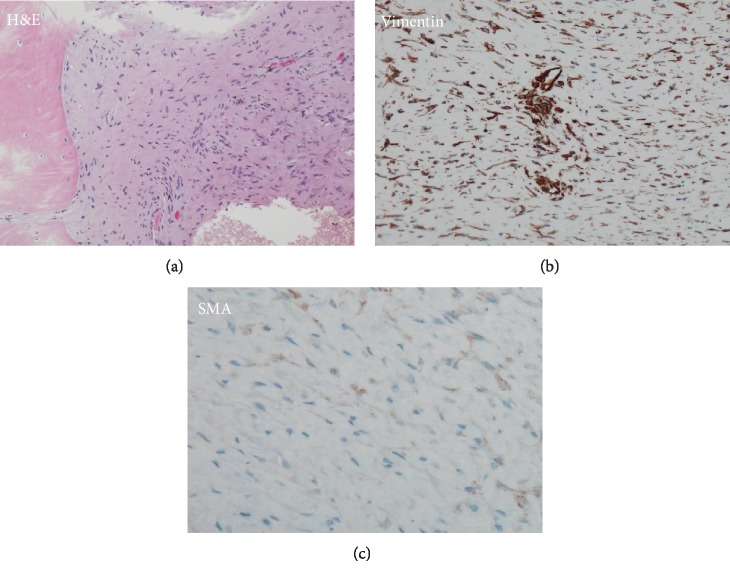
(a) The hematoxylin and eosin staining of specimen from the ilium showed the proliferation of spindle cells in an abundant collagenous matrix and involvement of tumor cells in the bone cortex, inducing bone destruction. (b, c) Immunohistochemically, the specimen was positive for cytoplasmic vimentin and smooth muscle action (SMA). (b) Vimentin. (c) SMA.

**Figure 3:**
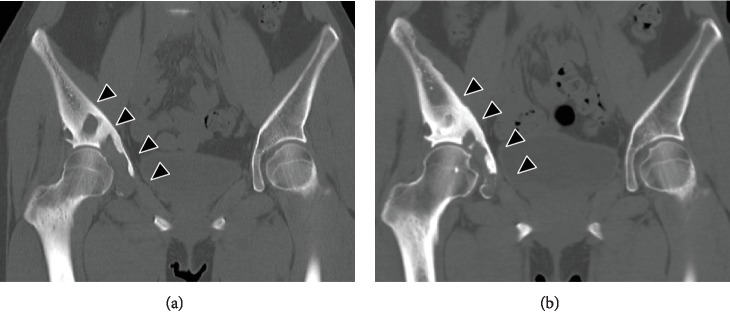
(a, b) Coronal view of computed tomography image at 10 years after the first visit revealed a partially sclerotic lesion of the ilium and acetabulum. (a) At first visit. (b) Ten years after the first visit.

A 21-year-old female patient presented to our orthopedic outpatient department with right hip pain. She had no history of preceding episodes of trauma or intense exercise. Her medical history was unremarkable. On physical examination, although the hip range of motion was normal, right hip pain occurred under loading. Blood laboratory findings were within normal limits. Radiography and computed tomography (CT) showed an osteolytic lesion of the right ilium and acetabulum with cortical destruction, marginal sclerosis, slight pseudotrabeculation, and bone expansion (Figures 1(a) and 1(b)). Magnetic resonance imaging (MRI) revealed a massive tumor lesion throughout the right ilium; T1- and T2-weighted images of the iliac lesion showed low-signal intensity compared to the muscle, indicative of fibrous tumor (Figures 1(c) and 1(d)). As the acetabulum was severely destroyed, there was a possibility of malignant tumor such as low-grade fibrosarcoma and malignant change of fibrous dysplasia. Open biopsy from the right ilium showed the proliferation of spindle cells in an abundant collagenous matrix and involvement of tumor cells in the bone cortex, inducing bone destruction (Figure 2(a)). Neither cellular atypia nor mitotic figures were observed. As the specimen in the lesion was a mature bone tissue and not C-shaped woven bone seen in fibrous dysplasia, the tumor was not considered as fibrous dysplasia. Furthermore, immunohistochemically, the specimen was positive for cytoplasmic vimentin and smooth muscle actin (SMA), suggesting a diagnosis of DFB (Figures 2(b) and 2(c)). However, as low-grade fibrosarcoma was not definitively excluded, close follow-up with radiological evaluation was performed. Although loading of the right leg was allowed, intense exercise such as sports was prohibited. Bisphosphonate was administered to delay lesion progression. The osteolytic lesion in the ilium changed to an osteosclerotic lesion over time, accompanied with a decrease in hip pain, allowing loading of the right leg. CT at 10 years after the first visit revealed a partially sclerotic lesion of the ilium and acetabulum (Figures 3(a) and 3(b)). In the 10 years of follow-up, the pain control was good.

## 3. Discussion

DFB is a benign, locally invasive bone tumor, initially described by Jaffe in 1958 [5]. The tumor often occurs in adolescents and young adults. DFB has a global annual morbidity of 2 to 4 in 1,000,000 and an etiology that has remained unclear [6]. The long bones are the most common sites of occurrence with mandibular, femoral, pelvic, radial, and tibial involvement in 23%, 15%, 13%, 12%, and 9% of cases, respectively.

Radiographically, DFB has been described as an osteolytic lesion with cortical bone destruction, marginal sclerosis, and pseudotrabeculation. On MRI, the lesion shows low-signal intensity on T1-weighted images with signal enhancement after contrast administration [7]. In the current study, radiography and CT showed an osteolytic lesion of the right ilium and acetabulum with cortical destruction, marginal sclerosis, slight pseudotrabeculation, and bone expansion, while MRI revealed low-signal intensity compared to muscle on T1- and T2-weighted images of a massive lesion present throughout the right ilium. These findings suggested a diagnosis of DFB. However, it is important to note the differential diagnoses obtained from radiographic findings including giant cell tumors, aneurysmal and solitary bone cysts, hemangioma, fibrous dysplasia, nonossifying fibroma, and chondromyxoid fibroma, and primary malignant lesions such as adamantinoma, fibrosarcoma, low-grade osteosarcoma, and metastatic carcinoma [8].

Histologically, DFB is composed of spindle cells with minimal cytological atypia and abundant collagen production, which is similar to a soft tissue desmoid tumor [5]. Immunohistochemically, DFB usually shares features identical to those of aggressive fibromatosis of the soft tissue, including positive b-catenin, vimentin, and SMA expression, whereas the expression of desmin, S-100, CD34, and MDM2 is negative [9]. In particular, important histological differential diagnoses of DFB include low-grade fibrosarcoma of the bone, fibrous dysplasia, and bony invasion of desmoid tumor, which is often the most difficult differential diagnosis to ascertain [10]. Compared with DFB, a typical fibrosarcoma is increasingly cellular with a herringbone pattern and exhibits increased polymorphisms and higher mitotic activity [11]. However, mitosis is not a prominent feature in low-grade bone fibrosarcoma. Furthermore, DFB has low morbidity and diagnosing this rare disease is challenging, with inaccurate diagnosis often among pathologists. Therefore, in such cases, close follow-up with radiological evaluation is needed. In the current case, histopathological examination showed proliferation of spindle cells in an abundant collagenous matrix without cellular atypia and mitotic figures and involvement of tumor cells in the bone cortex, inducing bone destruction. Furthermore, immunohistochemistry revealed positivity for cytoplasmic vimentin and SMA, strongly suggesting the possibility of DFB.

Treatment strategies for desmoplastic fibroma vary but may include curettage, wide resection with or without an allograft, cryosurgery, and amputation in recurrent cases. Böhm et al. analyzed 191 cases of DFB and reported that recurrence rates following curettage, excision, and wide resection were 55%, 72%, and 17%, respectively [4]. Consequently, it was considered that the recommended primary treatment for DFB is a marginal or wide resection of the tumors. Recently, although certain reports have described the successful treatment of DFB using curettage with lesion ablation [12], the follow-up duration in these studies was insufficient. Thus, although there are various surgical procedures for DFB, conservative therapy should be considered in cases where a massive lesion is located throughout the pelvis, including the acetabulum, as functional prognosis will be significantly worsened by wide resection. In the current case, although the tumor was present throughout the right ilium including the acetabulum, we first considered wide resection rather than curettage in terms of recurrence. However, as we expected that the functional prognosis will be worsened by wide resection of the ilium, conservative treatment was selected first, while surgery was reserved in the event of worsening pain.

There are 107 published cases of DFB in the English literature (mean follow-up periods: 46.3 months, range: 3-336 months). Of these, 9 report follow-up for over 10 years (mean follow-up periods: 179.4 months, range: 144-336 months). Table.  1 shows the sex, age, tumor location, treatment, and follow-up period of the 9 reports, suggesting that surgery was performed in all cases with long-term follow-up. However, there are no reports on follow-up for over 10 years after conservative treatment. Since there was no report of long-term follow-up after conservative treatment for DFB, careful medical examination with radiological evaluation was conducted once every 2 to 3 months. Although, currently, the present case is in the 10th year of follow-up following conservative treatment, the lesion of the ilium and acetabulum was converted from lytic to partially sclerotic, and the clinical course of the patient has been good with no pain. However, as DFB is locally aggressive and recurrent, careful follow-up is still required.

In conclusion, the present report described a 10-year follow-up for massive DFB of the ilium successfully treated without surgery.

## Figures and Tables

**Table 1:** Clinical data of the published reports of desmoplastic fibroma of the bone with over 10 years follow-up.

Reference	Sex	Age	Tumor location	Treatment	Follow-up period (month)
Evans et al. [13]	F	35	Proximal fibula	Excision	145
	F	22	Distal tibia	Amputation	158
	M	15	Radius	Excision	183
	M	24	Proximal tibia	Prosthesis replacement	181
Gong et al. [14]	F	66	Femur	Excision and internal fixation	336
Khatib and Pogrel [15]	F	8	Mandible	Supraperiosteal dissection	168
	F	9	Mandible	Segmental resection	156
	M	2	Mandible	Supraperiosteal dissection	144
Yokouchi et al. [11]	M	26	Femur	Curettage and bone grafting	144

The current case	F	21	Ilium	Conservative treatments	120
